# Finite time synchronization of memristor-based Cohen-Grossberg neural networks with mixed delays

**DOI:** 10.1371/journal.pone.0185007

**Published:** 2017-09-20

**Authors:** Chuan Chen, Lixiang Li, Haipeng Peng, Yixian Yang

**Affiliations:** 1 Information Security Center, State Key Laboratory of Networking and Switching Technology, Beijing University of Posts and Telecommunications, Beijing 100876, China; 2 State Key Laboratory of Public Big Data, Guizhou 550025, China; Lanzhou University of Technology, CHINA

## Abstract

Finite time synchronization, which means synchronization can be achieved in a settling time, is desirable in some practical applications. However, most of the published results on finite time synchronization don’t include delays or only include discrete delays. In view of the fact that distributed delays inevitably exist in neural networks, this paper aims to investigate the finite time synchronization of memristor-based Cohen-Grossberg neural networks (MCGNNs) with both discrete delay and distributed delay (mixed delays). By means of a simple feedback controller and novel finite time synchronization analysis methods, several new criteria are derived to ensure the finite time synchronization of MCGNNs with mixed delays. The obtained criteria are very concise and easy to verify. Numerical simulations are presented to demonstrate the effectiveness of our theoretical results.

## Introduction

**Fig 1:**
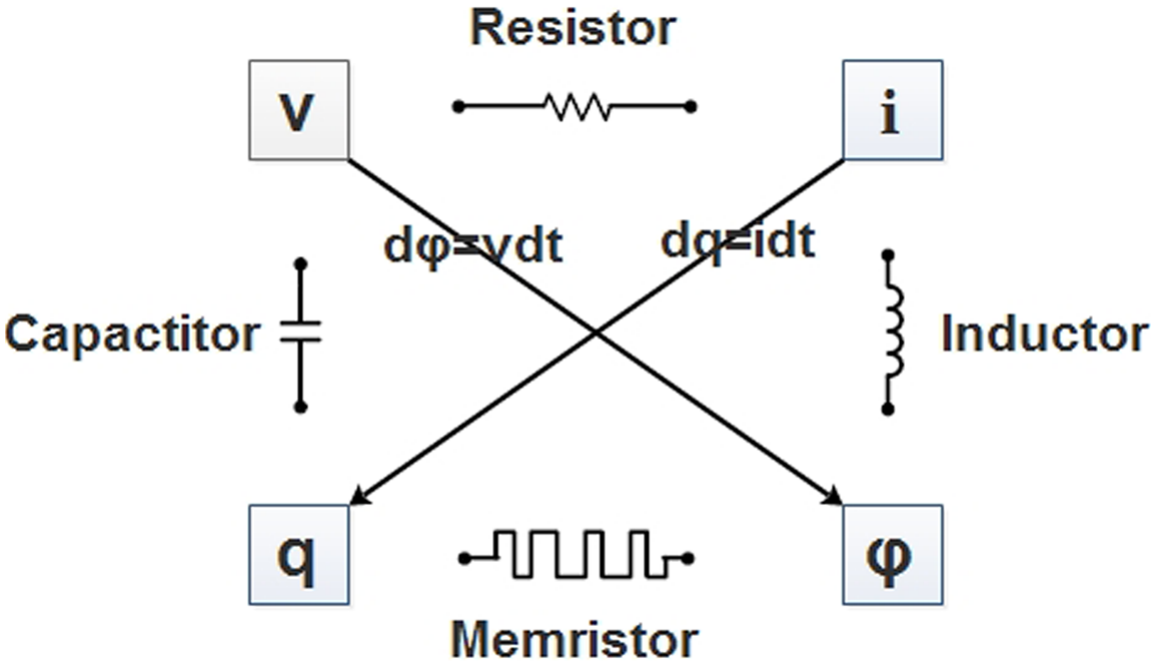
The relations among resistor (*R*), capacitor (*C*), inductor (*L*), memristor (*M*), voltage (*v*), current (*i*), charge (*q*) and flux (*φ*): *dv* = *Rdi*, *dq* = *Cdv*, *dφ* = *Ldi* and *dφ* = *Mdq*.

Memristor, which was first proposed by Chua in 1971 [1], is deemed as the fourth fundamental circuit element besides inductor, capacitor and resistor. In 2008, the prototype of memristor was first realized by the scientists of Hewlett-Packard (HP) [2]. Memristor, the contraction of memory resistor, reflects the nonlinear relationship between charge and flux (see Fig 1). It has been proved that memristor has variable resistance and the function of memory. In the artificial neural network, the synapses are usually modeled by resistors [3]. Since memristors own memory and perform more like real biological synapses, now memristors have been utilized to replace the resistors in artificial neural network to build memristor-based neural network (MNN), which is the appropriate candidate for simulating the human brain [4].

On the other hand, synchronization of complex networks [5–7] has received much attention due to its great application prospect in many different fields such as image encryption [8], secure communications [9] and associative memory [10]. By utilizing a memristor to replace the diode in Chuas circuit, Chua obtained several oscillators in [11]. Since then, various memristive chaotic systems have been proposed by using the similar methods. As we know, chaos [12, 13] presents complex nonlinear behaviours, but it can appears in a simple memristor-based Chuas circuit! So it is important to study the synchronization control of MNNs [14–17]. In [14], the exponential synchronization of MNNs with mixed delays was investigated via adaptive control. By means of intermittent control, the authors of [15] studied the stability and synchronization of memristor-based coupling neural networks with time-varying delays. Finite-time Mittag-Leffler synchronization of fractional-order memristive BAM neural networks with time delays was studied in [17]. Moreover, in view of the characteristics that signals transmit in real neural network, we should study the neural networks with time delays, including discrete delays [18, 19] and distributed delays [20, 21].

It should be pointed out that controller plays an important part in realizing synchronization. But how to design the optimal controller? It seems this problem has not been addressed. So far, many effective control methods have been proposed, such as the activation control [22], pinning control [23, 24], the linear separation method [25], the linear coupling method [26], impulsive control [27], adaptive control [14, 28], intermittent control [15], the sliding mode control [29], etc. However, in this paper, the controller that we design is a discontinuous feedback controller. Compared with the above-mentioned control methods, feedback control has the simplest form, and is very easy to be manipulated in practical applications.

In 1983, Cohen and Grossberg [30] proposed the Cohen-Grossberg neural network model, which is very general and important in all kinds of neural network models. Some important neural networks, such as cellular neural network and Hopfield neural network, can be deemed as the special cases of Cohen-Grossberg neural network. Although there have been many results about delayed Cohen-Grossberg neural networks [31–40], few of them are related to delayed MCGNNs. Recently, the exponential synchronization of MCGNNs with mixed delays was discussed in [41], the function projective synchronization of MCGNNs with time-varying discrete delays was studied in [42]. Obviously, most published results about synchronization control of MCGNNs only consider asymptotical synchronization and exponential synchronization, which mean that the synchronization time is infinite, but in application fields, it is more meaningful that the synchronization can be achieved in finite time. However, up to now, only Ref. [43] was concerning the finite time synchronization of MCGNNs with discrete delays. It should be pointed out that the distributed delays were not considered in [43], and the controller used in [43] was very complicated. As far as we know, there has been no published result on finite time synchronization of MCGNNs with mixed delays until now.

Inspired by the above analysis, this paper is devoted to studying the finite time synchronization problem of MCGNNs with mixed delays. The main contributions and originality of our paper are listed below: (i) This is the first attempt to investigate the finite time synchronization problem of MCGNNs with mixed delays, including time-varying discrete delays and distributed delays. Compared with the results in [43], the results in this paper are more general. (ii) The finite time synchronization analysis method used in this paper is a novel finite time synchronization analysis method, which has only been used in our another paper [44]. By adopting this novel analysis method, we derive some sufficient conditions that can ensure the finite time synchronization of the studied MCGNNs. Furthermore, the analysis method used in this paper can also be applied to analyze the finite time synchronization of other MNNs. (iii) In many literatures on the finite time synchronization of delayed systems, the controllers are very complicated. Although the neural network model considered in this paper is MCGNN with mixed delays, only simple feedback controllers are enough to derive the finite time synchronization of the studied MCGNNs. In some papers, the designed controllers were also similar to the controllers in this paper, however, only the asymptotical synchronization or the exponential synchronization of the studied systems can be obtained.

The rest of this paper is organized as follows. Some essential preliminaries are introduced in Section 2. In Section 3, our main results are derived. In Section 4, numerical simulations are presented to verify the theoretical results. Conclusions are drawn in Section 5.

## Preliminaries

Referring to some existing MCGNN models [41–43], in this paper, we consider the following MCGNN with mixed delays:

$$\begin{array}{ccc} {\dot{\xi }}_{i}\left(t\right) & = & -w_{i}\left(\xi_{i}\left(t\right)\right)[a_{i}\left(\xi_{i}\left(t\right)\right)-\sum_{j=1}^{n}b_{ij}\left(\xi_{i}\left(t\right)\right)f_{j}\left(\xi_{j}\left(t\right)\right)-\sum_{j=1}^{n}c_{ij}\left(\xi_{i}\left(t\right)\right)f_{j}\left(\xi_{j}\left(t-\tau_{ij}\left(t\right)\right)\right)\\ & & -\sum_{j=1}^{n}d_{ij}\left(\xi_{i}\left(t\right)\right)\int_{-\infty }^{t}K_{ij}\left(t-s\right)f_{j}\left(\xi_{j}\left(s\right)\right)ds-I_{i}],\;i=1,2,\ldots ,n, \end{array}$$ (1)

where *ξ*_*i*_(*t*) represents the state of the *i*th neuron; *w*_*i*_(⋅) is the amplification function; *a*_*i*_(⋅) denotes the appropriately behaved function; *τ*_*ij*_(*t*) is the discrete delay; *K*_*ij*_: [0, +∞) → [0, +∞) stands for the delay kernel of the unbounded distributed delay; *I*_*i*_ is the external input; the initial value of MCGNN (1) is *φ*(*s*) = (*φ*_1_(*s*), *φ*_2_(*s*), …, *φ*_*n*_(*s*))^*T*^, *s* ≤ 0; *b*_*ij*_(*ξ*_*i*_(*t*)), *c*_*ij*_(*ξ*_*i*_(*t*)) and *d*_*ij*_(*ξ*_*i*_(*t*)) are memristive connection weights, which are given by

$$\begin{array}{ccc} & & b_{ij}\left(\xi_{i}\left(t\right)\right)=\left\{ \begin{array}{c} b_{ij}^{\prime },\left|\xi_{i}\left(t\right)\right|\leq {ϒ}_{i},\\ b_{ij}^{\prime \prime },\left|\xi_{i}\left(t\right)\right|>{ϒ}_{i}, \end{array}\right.c_{ij}\left(\xi_{i}\left(t\right)\right)=\left\{ \begin{array}{c} c_{ij}^{\prime },\left|\xi_{i}\left(t\right)\right|\leq {ϒ}_{i},\\ c_{ij}^{\prime \prime },\left|\xi_{i}\left(t\right)\right|>{ϒ}_{i}, \end{array}\right.\\ & & d_{ij}\left(\xi_{i}\left(t\right)\right)=\left\{ \begin{array}{c} d_{ij}^{\prime },\left|\xi_{i}\left(t\right)\right|\leq {ϒ}_{i},\\ d_{ij}^{\prime \prime },\left|\xi_{i}\left(t\right)\right|>{ϒ}_{i}, \end{array}\right. \end{array}$$ (2)

for *i*, *j* = 1, 2, …, *n*, where ${ϒ}_{i},b_{ij}^{\prime },b_{ij}^{\prime \prime },c_{ij}^{\prime },c_{ij}^{\prime \prime },d_{ij}^{\prime },d_{ij}^{\prime \prime }$ are known constants [45, 46].

To derive the theoretical results, some assumptions will be needed:

(*A*_1_) 0 ≤ *τ*_*ij*_(*t*) ≤ *τ*_*ij*_, ${\dot{\tau }}_{ij}\left(t\right)\leq \sigma_{ij}<1$, where *τ*_*ij*_ > 0 and *σ*_*ij*_ > 0 are constants, *i*, *j* = 1, 2, …, *n*.

(*A*_2_) *w*_*i*_(⋅) is continuous and $0<\underline{w_{i}}\leq w_{i}\left(\cdot \right)\leq \bar{w_{i}}$, where $\underline{w_{i}}>0$ and $\bar{w_{i}}>0$ are constants, *i* = 1, 2, …, *n*.

(*A*_3_) ${\dot{a}}_{i}\left(\cdot \right)\geq a_{i}$, where *a*_*i*_ > 0 are constants, *i* = 1, 2, …, *n*.

(*A*_4_) For ∀*x*, *y* ∈ *R*, there exist constants *l*_*i*_ > 0 such that

$$\begin{array}{c} |f_{i}\left(x\right)-f_{i}\left(y\right)|\leq l_{i}\left|x-y\right|,\;i=1,2,\ldots ,n. \end{array}$$

(*A*_5_) There exist constants *M*_*i*_ > 0 such that |*f*_*i*_(⋅)| ≤ *M*_*i*_, *i* = 1, 2, …, *n*.

(*A*_6_) There exist constants *K*_*ij*_ > 0 such that

$$\begin{array}{c} \int_{0}^{+\infty }K_{ij}\left(s\right)ds\leq K_{ij},\;i,j=1,2,\ldots ,n. \end{array}$$

Choose a transformation function Φ_*i*_(⋅), which satisfies

$$\begin{array}{c} \frac{d}{du}\left(\Phi_{i}\left(u\right)\right)=\frac{1}{w_{i}\left(u\right)}. \end{array}$$ (3)

In view of $\frac{1}{w_{i}\left(u\right)}>0$, we know Φ_*i*_(⋅) is strictly monotone increasing, then $\Phi_{i}^{-1}\left(\cdot \right)$ exists. Let *x*_*i*_(*t*) = Φ_*i*_(*ξ*_*i*_(*t*)), we have ${\dot{x}}_{i}\left(t\right)=\frac{d\Phi_{i}\left(\xi_{i}\left(t\right)\right)}{\xi_{i}\left(t\right)}{\dot{\xi }}_{i}\left(t\right)=\frac{1}{w_{i}\left(\xi_{i}\left(t\right)\right)}{\dot{\xi }}_{i}\left(t\right),$ $\xi_{i}\left(t\right)=\Phi_{i}^{-1}\left(x_{i}\left(t\right)\right)$. On the other hand, by the derivative theorem of inverse function, $\frac{d}{du}\left(\Phi_{i}^{-1}\left(u\right)\right)=w_{i}\left(u\right)$. Then it follows that

$$\begin{array}{ccc} {\dot{x}}_{i}\left(t\right) & = & -a_{i}\left(\Phi_{i}^{-1}\left(x_{i}\left(t\right)\right)\right)+\sum_{j=1}^{n}b_{ij}\left(\Phi_{i}^{-1}\left(x_{i}\left(t\right)\right)\right)f_{j}\left(\Phi_{j}^{-1}\left(x_{j}\left(t\right)\right)\right)\\ & & +\sum_{j=1}^{n}c_{ij}\left(\Phi_{i}^{-1}\left(x_{i}\left(t\right)\right)\right)f_{j}\left(\Phi_{j}^{-1}\left(x_{j}\left(t-\tau_{ij}\left(t\right)\right)\right)\right)\\ & & +\sum_{j=1}^{n}d_{ij}\left(\Phi_{i}^{-1}\left(x_{i}\left(t\right)\right)\right)\int_{-\infty }^{t}K_{ij}\left(t-s\right)f_{j}\left(\Phi_{j}^{-1}\left(x_{j}\left(s\right)\right)\right)ds+I_{i},\;i=1,2,\ldots ,n. \end{array}$$ (4)

Throughout this paper, we set ${\bar{b}}_{ij}=\max\left\{b_{ij}^{\prime },b_{ij}^{\prime \prime }\right\},{\underline{b}}_{ij}=\min\left\{b_{ij}^{\prime },b_{ij}^{\prime \prime }\right\},b_{ij}^{u}=\max\{|b_{ij}^{\prime }\left|,\right|b_{ij}^{\prime \prime }\left|\right\}$, ${\bar{c}}_{ij}=\max\left\{c_{ij}^{\prime },c_{ij}^{\prime \prime }\right\},{\underline{c}}_{ij}=\min\left\{c_{ij}^{\prime },c_{ij}^{\prime \prime }\right\},c_{ij}^{u}=\max\{|c_{ij}^{\prime }\left|,\right|c_{ij}^{\prime \prime }\left|\right\}$, ${\bar{d}}_{ij}=\max\left\{d_{ij}^{\prime },d_{ij}^{\prime \prime }\right\},{\underline{d}}_{ij}=\min\left\{d_{ij}^{\prime },d_{ij}^{\prime \prime }\right\},d_{ij}^{u}=\max\{|d_{ij}^{\prime }\left|,\right|d_{ij}^{\prime \prime }\left|\right\}$, for *i*, *j* = 1, 2, …, *n*. $\bar{co}\left[E\right]$ stands for the closure of the convex hull generated by set *E*.

Based on the relevant theories of differential inclusions and set-valued maps [47, 48], we can derive that:

$$\begin{array}{ccc} & & {\dot{x}}_{i}\left(t\right)\in -a_{i}\left(\Phi_{i}^{-1}\left(x_{i}\left(t\right)\right)\right)+\sum_{j=1}^{n}\bar{co}\left[b_{ij}\left(\Phi_{i}^{-1}\left(x_{i}\left(t\right)\right)\right)\right]f_{j}\left(\Phi_{j}^{-1}\left(x_{j}\left(t\right)\right)\right)\\ & & \;+\sum_{j=1}^{n}\bar{co}\left[c_{ij}\left(\Phi_{i}^{-1}\left(x_{i}\left(t\right)\right)\right)\right]f_{j}\left(\Phi_{j}^{-1}\left(x_{j}\left(t-\tau_{ij}\left(t\right)\right)\right)\right)\\ & & \;+\sum_{j=1}^{n}\bar{co}\left[d_{ij}\left(\Phi_{i}^{-1}\left(x_{i}\left(t\right)\right)\right)\right]\int_{-\infty }^{t}K_{ij}\left(t-s\right)f_{j}\left(\Phi_{j}^{-1}\left(x_{j}\left(s\right)\right)\right)ds+I_{i},\;i=1,2,\ldots ,n, \end{array}$$ (5)

where

$$\begin{array}{ccc} \bar{co}\left[b_{ij}\left(\Phi_{i}^{-1}\left(x_{i}\left(t\right)\right)\right)\right] & = & \left\{ \begin{array}{cc} b_{ij}^{\prime }, & |\Phi_{i}^{-1}\left(x_{i}\left(t\right)\right)|<{ϒ}_{i},\\ {\underline{b}}_{ij},{\bar{b}}_{ij}], & |\Phi_{i}^{-1}\left(x_{i}\left(t\right)\right)|={ϒ}_{i},\\ b_{ij}^{\prime \prime }, & |\Phi_{i}^{-1}\left(x_{i}\left(t\right)\right)|>{ϒ}_{i}, \end{array}\right.\\ \bar{co}\left[c_{ij}\left(\Phi_{i}^{-1}\left(x_{i}\left(t\right)\right)\right)\right] & = & \left\{ \begin{array}{cc} c_{ij}^{\prime }, & |\Phi_{i}^{-1}\left(x_{i}\left(t\right)\right)|<{ϒ}_{i},\\ {\underline{c}}_{ij},{\bar{c}}_{ij}], & |\Phi_{i}^{-1}\left(x_{i}\left(t\right)\right)|={ϒ}_{i},\\ c_{ij}^{\prime \prime }, & |\Phi_{i}^{-1}\left(x_{i}\left(t\right)\right)|>{ϒ}_{i}, \end{array}\right.\\ \bar{co}\left[d_{ij}\left(\Phi_{i}^{-1}\left(x_{i}\left(t\right)\right)\right)\right] & = & \left\{ \begin{array}{cc} d_{ij}^{\prime }, & |\Phi_{i}^{-1}\left(x_{i}\left(t\right)\right)|<{ϒ}_{i},\\ {\underline{d}}_{ij},{\bar{d}}_{ij}], & |\Phi_{i}^{-1}\left(x_{i}\left(t\right)\right)|={ϒ}_{i},\\ d_{ij}^{\prime \prime }, & |\Phi_{i}^{-1}\left(x_{i}\left(t\right)\right)|>{ϒ}_{i}, \end{array}\right. \end{array}$$ (6)

for *i*, *j* = 1, 2, …, *n*. Then, there exist ${\overset{´}{b}}_{ij}\left(t\right)\in \bar{co}\left[b_{ij}\left(\Phi_{i}^{-1}\left(x_{i}\left(t\right)\right)\right)\right]$, ${\overset{´}{c}}_{ij}\left(t\right)\in \bar{co}\left[c_{ij}\left(\Phi_{i}^{-1}\left(x_{i}\left(t\right)\right)\right)\right]$ and ${\overset{´}{d}}_{ij}\left(t\right)\in \bar{co}\left[d_{ij}\left(\Phi_{i}^{-1}\left(x_{i}\left(t\right)\right)\right)\right]$ such that

$$\begin{array}{ccc} {\dot{x}}_{i}\left(t\right) & = & -a_{i}\left(\Phi_{i}^{-1}\left(x_{i}\left(t\right)\right)\right)+\sum_{j=1}^{n}{\overset{´}{b}}_{ij}\left(t\right)f_{j}\left(\Phi_{j}^{-1}\left(x_{j}\left(t\right)\right)\right)+\sum_{j=1}^{n}{\overset{´}{c}}_{ij}\left(t\right)f_{j}\left(\Phi_{j}^{-1}\left(x_{j}\left(t-\tau_{ij}\left(t\right)\right)\right)\right)\\ & & +\sum_{j=1}^{n}{\overset{´}{d}}_{ij}\left(t\right)\int_{-\infty }^{t}K_{ij}\left(t-s\right)f_{j}\left(\Phi_{j}^{-1}\left(x_{j}\left(s\right)\right)\right)ds+I_{i},\;i=1,2,\ldots ,n. \end{array}$$ (7)

MCGNN (1) is referred to as the drive system, this is the corresponding response system:

$$\begin{array}{ccc} {\dot{\eta }}_{i}\left(t\right) & = & -w_{i}\left(\eta_{i}\left(t\right)\right)[a_{i}\left(\eta_{i}\left(t\right)\right)-\sum_{j=1}^{n}b_{ij}\left(\eta_{i}\left(t\right)\right)f_{j}\left(\eta_{j}\left(t\right)\right)-\sum_{j=1}^{n}c_{ij}\left(\eta_{i}\left(t\right)\right)f_{j}\left(\eta_{j}\left(t-\tau_{ij}\left(t\right)\right)\right)\\ & & -\sum_{j=1}^{n}d_{ij}\left(\eta_{i}\left(t\right)\right)\int_{-\infty }^{t}K_{ij}\left(t-s\right)f_{j}\left(\eta_{j}\left(s\right)\right)ds-I_{i}]+R_{i}\left(t\right),\;i=1,2,\ldots ,n, \end{array}$$ (8)

where *R*_*i*_(*t*) is the appropriate controller; the initial value of MCGNN (8) is *ϕ*(*s*) = (*ϕ*_1_(*s*), *ϕ*_2_(*s*), …, *ϕ*_*n*_(*s*))^*T*^, *s* ≤ 0; *b*_*ij*_(*η*_*i*_(*t*)), *c*_*ij*_(*η*_*i*_(*t*)) and *d*_*ij*_(*η*_*i*_(*t*)) are defined as:

$$\begin{array}{ccc} b_{ij}\left(\eta_{i}\left(t\right)\right) & = & \left\{ \begin{array}{cc} b_{ij}^{\prime }, & |\eta_{i}\left(t\right)|\leq {ϒ}_{i},\\ b_{ij}^{\prime \prime }, & |\eta_{i}\left(t\right)|>{ϒ}_{i}, \end{array}\right.c_{ij}\left(\eta_{i}\left(t\right)\right)=\left\{ \begin{array}{cc} c_{ij}^{\prime }, & |\eta_{i}\left(t\right)|\leq {ϒ}_{i},\\ c_{ij}^{\prime \prime }, & |\eta_{i}\left(t\right)|>{ϒ}_{i}, \end{array}\right.\\ d_{ij}\left(\eta_{i}\left(t\right)\right) & = & \left\{ \begin{array}{cc} d_{ij}^{\prime }, & |\eta_{i}\left(t\right)|\leq {ϒ}_{i},\\ d_{ij}^{\prime \prime }, & |\eta_{i}\left(t\right)|>{ϒ}_{i}, \end{array}\right. \end{array}$$ (9)

for *i*, *j* = 1, 2, …, *n*.

In this paper, we design such a feedback controller:

$$\begin{array}{c} R_{i}\left(t\right)=-p_{i}\left(\eta_{i}\left(t\right)-\xi_{i}\left(t\right)\right)-q_{i}sign\left(\eta_{i}\left(t\right)-\xi_{i}\left(t\right)\right),\;i=1,2,\ldots ,n, \end{array}$$ (10)

where *p*_*i*_ > 0 and *q*_*i*_ > 0 are control gains.

Similarly, it can be derived that

$$\begin{array}{ccc} {\dot{y}}_{i}\left(t\right) & = & -a_{i}\left(\Phi_{i}^{-1}\left(y_{i}\left(t\right)\right)\right)+\sum_{j=1}^{n}{\overset{`}{b}}_{ij}\left(t\right)f_{j}\left(\Phi_{j}^{-1}\left(y_{j}\left(t\right)\right)\right)+\sum_{j=1}^{n}{\overset{`}{c}}_{ij}\left(t\right)f_{j}\left(\Phi_{j}^{-1}\left(y_{j}\left(t-\tau_{ij}\left(t\right)\right)\right)\right)\\ & & +\sum_{j=1}^{n}{\overset{`}{d}}_{ij}\left(t\right)\int_{-\infty }^{t}K_{ij}\left(t-s\right)f_{j}\left(\Phi_{j}^{-1}\left(y_{j}\left(s\right)\right)\right)ds+I_{i}\\ & & -\frac{p_{i}}{w_{i}\left(\Phi_{i}^{-1}\left(y_{i}\left(t\right)\right)\right)}\left(\Phi_{i}^{-1}\left(y_{i}\left(t\right)\right)-\Phi_{i}^{-1}\left(x_{i}\left(t\right)\right)\right)\\ & & -\frac{q_{i}}{w_{i}\left(\Phi_{i}^{-1}\left(y_{i}\left(t\right)\right)\right)}sign\left(\Phi_{i}^{-1}\left(y_{i}\left(t\right)\right)-\Phi_{i}^{-1}\left(x_{i}\left(t\right)\right)\right),\;i=1,2,\ldots ,n, \end{array}$$ (11)

where *y*_*i*_(*t*) = Φ_*i*_(*η*_*i*_(*t*)), ${\overset{`}{b}}_{ij}\left(t\right)\in \bar{co}\left[b_{ij}\left(\Phi_{i}^{-1}\left(y_{i}\left(t\right)\right)\right)\right]$, ${\overset{`}{c}}_{ij}\left(t\right)\in \bar{co}\left[c_{ij}\left(\Phi_{i}^{-1}\left(y_{i}\left(t\right)\right)\right)\right]$, ${\overset{`}{d}}_{ij}\left(t\right)\in \bar{co}\left[d_{ij}\left(\Phi_{i}^{-1}\left(y_{i}\left(t\right)\right)\right)\right]$ and

$$\begin{array}{ccc} \bar{co}\left[b_{ij}\left(\Phi_{i}^{-1}\left(y_{i}\left(t\right)\right)\right)\right] & = & \left\{ \begin{array}{cc} b_{ij}^{\prime }, & |\Phi_{i}^{-1}\left(y_{i}\left(t\right)\right)|<{ϒ}_{i},\\ {\underline{b}}_{ij},{\bar{b}}_{ij}], & |\Phi_{i}^{-1}\left(y_{i}\left(t\right)\right)|={ϒ}_{i},\\ b_{ij}^{\prime \prime }, & |\Phi_{i}^{-1}\left(y_{i}\left(t\right)\right)|>{ϒ}_{i}, \end{array}\right.\\ \bar{co}\left[c_{ij}\left(\Phi_{i}^{-1}\left(y_{i}\left(t\right)\right)\right)\right] & = & \left\{ \begin{array}{cc} c_{ij}^{\prime }, & |\Phi_{i}^{-1}\left(y_{i}\left(t\right)\right)|<{ϒ}_{i},\\ {\underline{c}}_{ij},{\bar{c}}_{ij}], & |\Phi_{i}^{-1}\left(y_{i}\left(t\right)\right)|={ϒ}_{i},\\ c_{ij}^{\prime \prime }, & |\Phi_{i}^{-1}\left(y_{i}\left(t\right)\right)|>{ϒ}_{i}, \end{array}\right.\\ \bar{co}\left[d_{ij}\left(\Phi_{i}^{-1}\left(y_{i}\left(t\right)\right)\right)\right] & = & \left\{ \begin{array}{cc} d_{ij}^{\prime }, & |\Phi_{i}^{-1}\left(y_{i}\left(t\right)\right)|<{ϒ}_{i},\\ {\underline{d}}_{ij},{\bar{d}}_{ij}], & |\Phi_{i}^{-1}\left(y_{i}\left(t\right)\right)|={ϒ}_{i},\\ d_{ij}^{\prime \prime }, & |\Phi_{i}^{-1}\left(y_{i}\left(t\right)\right)|>{ϒ}_{i}, \end{array}\right. \end{array}$$ (12)

for *i*, *j* = 1, 2, …, *n*.

Let *e*_*i*_(*t*) = *y*_*i*_(*t*) − *x*_*i*_(*t*), *i* = 1, 2, …, *n*, then we have

$$\begin{array}{ccc} {\dot{e}}_{i}\left(t\right) & = & -[a_{i}\left(\Phi_{i}^{-1}\left(y_{i}\left(t\right)\right)\right)-a_{i}\left(\Phi_{i}^{-1}\left(x_{i}\left(t\right)\right)\right)]\\ & & +\sum_{j=1}^{n}{\overset{`}{b}}_{ij}\left(t\right)\left[f_{j}\left(\Phi_{j}^{-1}\left(y_{j}\left(t\right)\right)\right)-f_{j}\left(\Phi_{j}^{-1}\left(x_{j}\left(t\right)\right)\right)\right]\\ & & +\sum_{j=1}^{n}{\overset{`}{c}}_{ij}\left(t\right)\left[f_{j}\left(\Phi_{j}^{-1}\left(y_{j}\left(t-\tau_{ij}\left(t\right)\right)\right)\right)-f_{j}\left(\Phi_{j}^{-1}\left(x_{j}\left(t-\tau_{ij}\left(t\right)\right)\right)\right)\right]\\ & & +\sum_{j=1}^{n}{\overset{`}{d}}_{ij}\left(t\right)\int_{-\infty }^{t}K_{ij}\left(t-s\right)\left[f_{j}\left(\Phi_{j}^{-1}\left(y_{j}\left(s\right)\right)\right)-f_{j}\left(\Phi_{j}^{-1}\left(x_{j}\left(s\right)\right)\right)\right]ds\\ & & +\sum_{j=1}^{n}\left({\overset{`}{b}}_{ij}\left(t\right)-{\overset{´}{b}}_{ij}\left(t\right)\right)f_{j}(\Phi_{j}^{-1}\left(x_{j}\left(t\right)\right)+\sum_{j=1}^{n}\left({\overset{`}{c}}_{ij}\left(t\right)-{\overset{´}{c}}_{ij}\left(t\right)\right)f_{j}(\Phi_{j}^{-1}\left(x_{j}\left(t-\tau_{ij}\left(t\right)\right)\right)\\ & & +\sum_{j=1}^{n}\left({\overset{`}{d}}_{ij}\left(t\right)-{\overset{´}{d}}_{ij}\left(t\right)\right)\int_{-\infty }^{t}K_{ij}\left(t-s\right)f_{j}\left(\Phi_{j}^{-1}\left(x_{j}\left(s\right)\right)\right)ds\\ & & -\frac{p_{i}}{w_{i}\left(\Phi_{i}^{-1}\left(y_{i}\left(t\right)\right)\right)}\left(\Phi_{i}^{-1}\left(y_{i}\left(t\right)\right)-\Phi_{i}^{-1}\left(x_{i}\left(t\right)\right)\right)\\ & & -\frac{q_{i}}{w_{i}\left(\Phi_{i}^{-1}\left(y_{i}\left(t\right)\right)\right)}sign\left(\Phi_{i}^{-1}\left(y_{i}\left(t\right)\right)-\Phi_{i}^{-1}\left(x_{i}\left(t\right)\right)\right),\;i=1,2,\ldots ,n, \end{array}$$ (13)

with initial value *ψ*(*s*) = (*ψ*_1_(*s*), *ψ*_2_(*s*), …, *ψ*_*n*_(*s*))^*T*^, *s* ≤ 0, where *ψ*_*i*_(*s*) = Φ_*i*_(*ϕ*_*i*_(*s*)) − Φ_*i*_(*φ*_*i*_(*s*)), *i* = 1, 2, …, *n*.

**Lemma 1** [49]. (Chain Rule) If *V*(⋅) : *R*^*n*^ → *R* is C−regular and *x*(*t*) ∈ *R*^*n*^ is absolutely continuous on any compact subinterval of [0, +∞), then *V*(*x*(*t*)): [0, +∞) → *R* is differentiable for *a*.*e*.*t* ∈ [0, +∞) and

$$\begin{array}{c} \frac{d}{dt}V\left(x\left(t\right)\right)=v\left(t\right)\dot{x}\left(t\right),\;\forall v\left(t\right)\in \partial V\left(x\left(t\right)\right). \end{array}$$

where ∂*V*(*x*(*t*)) is the Clarke generalized gradient.

**Definition 1**. MCGNN (8) is said to be synchronized with MCGNN (1) in finite time, if there exists a constant t* > 0 such that $\lim_{t\to t^{*}}e_{i}\left(t\right)=0$ and *e*_*i*_(*t*) ≡ 0 for *t* ≥ t*, *i* = 1, 2, …, *n*, where t* is called the settling time.

**Remark 1**. $\lim_{t\to t^{*}}e_{i}\left(t\right)=0$ and *e*_*i*_(*t*) ≡ 0 for *t* ≥ t* mean that $\lim_{t\to t^{*}}x_{i}\left(t\right)=\lim_{t\to t^{*}}y_{i}\left(t\right)$ and *x*_*i*_(*t*) ≡ *y*_*i*_(*t*) for *t* ≥ t*, that is to say, $\lim_{t\to t^{*}}\Phi_{i}\left(\xi_{i}\left(t\right)\right)=\lim_{t\to t^{*}}\Phi_{i}\left(\eta_{i}\left(t\right)\right)$ and Φ_*i*_(*ξ*_*i*_(*t*)) ≡ Φ_*i*_(*η*_*i*_(*t*)) for *t* ≥ t*. Since Φ_*i*_(⋅) is strictly monotone increasing, we know $\lim_{t\to t^{*}}e_{i}\left(t\right)=0$ and *e*_*i*_(*t*) ≡ 0 for *t* ≥ t* are also equivalent to $\lim_{t\to t^{*}}\xi_{i}\left(t\right)=\lim_{t\to t^{*}}\eta_{i}\left(t\right)$ and *ξ*_*i*_(*t*) ≡ *η*_*i*_(*t*) for *t* ≥ t*.

## Main results

In this section, we will derive some sufficient conditions that can guarantee the finite time synchronization of MCGNNs (1) and (8).

**Theorem 1**. Let assumptions *A*_1_-*A*_6_ hold. If control gains *p*_*i*_ and *q*_*i*_ satisfy

$$\begin{array}{ccc} p_{i} & \geq & -{\bar{w}}_{i}a_{i}+\frac{{\bar{w}}_{i}^{2}l_{i}}{{\underline{w}}_{i}}\sum_{j=1}^{n}\left(b_{ji}^{u}+\frac{1}{1-\sigma_{ji}}c_{ji}^{u}+d_{ji}^{u}K_{ji}\right),\\ q_{i} & > & {\bar{w}}_{i}\sum_{j=1}^{n}\left({\bar{b}}_{ij}-{\underline{b}}_{ij}+{\bar{c}}_{ij}-{\underline{c}}_{ij}+{\bar{d}}_{ij}K_{ij}-{\underline{d}}_{ij}K_{ij}\right)M_{j},\;i=1,2,\ldots ,n, \end{array}$$ (14)

MCGNN (8) will be synchronized with MCGNN (1) in finite time under the controller (10).

**Proof**. We design such a Lyapunov function:

$$\begin{array}{c} V\left(t\right)=V_{1}\left(t\right)+V_{2}\left(t\right)+V_{3}\left(t\right), \end{array}$$ (15)

where

$$\begin{array}{ccc} V_{1}\left(t\right) & = & \sum_{i=1}^{n}\left|e_{i}\left(t\right)\right|,\\ V_{2}\left(t\right) & = & \sum_{i=1}^{n}\sum_{j=1}^{n}\frac{1}{1-\sigma_{ij}}c_{ij}^{u}l_{j}{\bar{w}}_{j}\int_{t-\tau_{ij}\left(t\right)}^{t}\left|e_{j}\left(z\right)\right|dz,\\ V_{3}\left(t\right) & = & \sum_{i=1}^{n}\sum_{j=1}^{n}d_{ij}^{u}l_{j}{\bar{w}}_{j}\int_{-\infty }^{0}\int_{t+s}^{t}K_{ij}\left(-s\right)\left|e_{j}\left(z\right)\right|dzds. \end{array}$$ (16)

By Lemma 1, the derivative of *V*_1_(*t*) can be calculated as:

$$\begin{array}{ccc} {\dot{V}}_{1}\left(t\right) & = & \sum_{i=1}^{n}signe_{i}\left(t\right)\{-[a_{i}\left(\Phi_{i}^{-1}\left(y_{i}\left(t\right)\right)\right)-a_{i}\left(\Phi_{i}^{-1}\left(x_{i}\left(t\right)\right)\right)]\\ & & +\sum_{j=1}^{n}{\overset{`}{b}}_{ij}\left(t\right)\left[f_{j}\left(\Phi_{j}^{-1}\left(y_{j}\left(t\right)\right)\right)-f_{j}\left(\Phi_{j}^{-1}\left(x_{j}\left(t\right)\right)\right)\right]\\ & & +\sum_{j=1}^{n}{\overset{`}{c}}_{ij}\left(t\right)\left[f_{j}\left(\Phi_{j}^{-1}\left(y_{j}\left(t-\tau_{ij}\left(t\right)\right)\right)\right)-f_{j}\left(\Phi_{j}^{-1}\left(x_{j}\left(t-\tau_{ij}\left(t\right)\right)\right)\right)\right]\\ & & +\sum_{j=1}^{n}{\overset{`}{d}}_{ij}\left(t\right)\int_{-\infty }^{t}K_{ij}\left(t-s\right)\left[f_{j}\left(\Phi_{j}^{-1}\left(y_{j}\left(s\right)\right)\right)-f_{j}\left(\Phi_{j}^{-1}\left(x_{j}\left(s\right)\right)\right)\right]ds\\ & & +\sum_{j=1}^{n}\left({\overset{`}{b}}_{ij}\left(t\right)-{\overset{´}{b}}_{ij}\left(t\right)\right)f_{j}\left(\Phi_{j}^{-1}\left(x_{j}\left(t\right)\right)\right)+\sum_{j=1}^{n}\left({\overset{`}{c}}_{ij}\left(t\right)-{\overset{´}{c}}_{ij}\left(t\right)\right)f_{j}\left(\Phi_{j}^{-1}\left(x_{j}\left(t-\tau_{ij}\left(t\right)\right)\right)\right)\\ & & +\sum_{j=1}^{n}\left({\overset{`}{d}}_{ij}\left(t\right)-{\overset{´}{d}}_{ij}\left(t\right)\right)\int_{-\infty }^{t}K_{ij}\left(t-s\right)f_{j}\left(\Phi_{j}^{-1}\left(x_{j}\left(s\right)\right)\right)ds\\ & & -\frac{p_{i}}{w_{i}\left(\Phi_{i}^{-1}\left(y_{i}\left(t\right)\right)\right)}\left(\Phi_{i}^{-1}\left(y_{i}\left(t\right)\right)-\Phi_{i}^{-1}\left(x_{i}\left(t\right)\right)\right)\\ & & -\frac{q_{i}}{w_{i}\left(\Phi_{i}^{-1}\left(y_{i}\left(t\right)\right)\right)}sign\left(\Phi_{i}^{-1}\left(y_{i}\left(t\right)\right)-\Phi_{i}^{-1}\left(x_{i}\left(t\right)\right)\right)\}. \end{array}$$ (17)

Based on assumptions *A*_2_ and *A*_3_, it can be obtained that

$$\begin{array}{ccc} & & signe_{i}\left(t\right)\left\{-[a_{i}\left(\Phi_{i}^{-1}\left(y_{i}\left(t\right)\right)\right)-a_{i}\left(\Phi_{i}^{-1}\left(x_{i}\left(t\right)\right)\right)]\right\}\\ & & \;=-signe_{i}\left(t\right)a_{i}^{\prime }\left(\theta_{1}\right)\left[\Phi_{i}^{-1}\left(y_{i}\left(t\right)\right)-\Phi_{i}^{-1}\left(x_{i}\left(t\right)\right)\right]\\ & & \;=-signe_{i}\left(t\right)a_{i}^{\prime }\left(\theta_{1}\right)\left({\left(\Phi_{i}^{-1}\right)}^{\prime }\left(\theta_{2}\right)\right)e_{i}\left(t\right)\leq -a_{i}{\underline{w}}_{i}\left|e_{i}\left(t\right)\right|, \end{array}$$ (18)

where *θ*_1_ is between $\Phi_{i}^{-1}\left(y_{i}\left(t\right)\right)$ and $\Phi_{i}^{-1}\left(x_{i}\left(t\right)\right)$, *θ*_2_ is between *y*_*i*_(*t*) and *x*_*i*_(*t*).

Based on assumptions *A*_2_ and *A*_4_, it follows that

$$\begin{array}{ccc} & & signe_{i}\left(t\right){\overset{`}{b}}_{ij}\left(t\right)\left[f_{j}\left(\Phi_{j}^{-1}\left(y_{j}\left(t\right)\right)\right)-f_{j}\left(\Phi_{j}^{-1}\left(x_{j}\left(t\right)\right)\right)\right]\\ & & \;\leq |{\overset{`}{b}}_{ij}\left(t\right)|\cdot l_{j}|\Phi_{j}^{-1}\left(y_{j}\left(t\right)\right)-\Phi_{j}^{-1}\left(x_{j}\left(t\right)\right)|\leq b_{ij}^{u}l_{j}{\bar{w}}_{j}\left|e_{j}\left(t\right)\right|. \end{array}$$ (19)

Similarly, we have

$$\begin{array}{ccc} & & signe_{i}\left(t\right){\overset{`}{c}}_{ij}\left(t\right)\left[f_{j}\left(\Phi_{j}^{-1}\left(y_{j}\left(t-\tau_{ij}\left(t\right)\right)\right)\right)-f_{j}\left(\Phi_{j}^{-1}\left(x_{j}\left(t-\tau_{ij}\left(t\right)\right)\right)\right)\right]\\ & & \;\leq c_{ij}^{u}l_{j}{\bar{w}}_{j}\left|e_{j}\left(t-\tau_{ij}\left(t\right)\right)\right| \end{array}$$ (20)

and

$$\begin{array}{ccc} & & signe_{i}\left(t\right){\overset{`}{d}}_{ij}\left(t\right)\int_{-\infty }^{t}K_{ij}\left(t-s\right)\left[f_{j}\left(\Phi_{j}^{-1}\left(y_{j}\left(s\right)\right)\right)-f_{j}\left(\Phi_{j}^{-1}\left(x_{j}\left(s\right)\right)\right)\right]ds\\ & & \;\leq d_{ij}^{u}l_{j}{\bar{w}}_{j}\int_{-\infty }^{t}K_{ij}\left(t-s\right)\left|e_{j}\left(s\right)\right|ds. \end{array}$$ (21)

Based on assumption *A*_5_, it follows that

$$\begin{array}{c} signe_{i}\left(t\right)\left({\overset{`}{b}}_{ij}\left(t\right)-{\overset{´}{b}}_{ij}\left(t\right)\right)f_{j}(\Phi_{j}^{-1}\left(x_{j}\left(t\right)\right)\leq \left|{\overset{`}{b}}_{ij}\left(t\right)-{\overset{´}{b}}_{ij}\left(t\right)\right|M_{j}\gamma_{i}\leq \left({\bar{b}}_{ij}-{\underline{b}}_{ij}\right)M_{j}\gamma_{i}, \end{array}$$ (22)

where *γ*_*i*_ = 0 if *e*_*i*_(*t*) = 0, otherwise *γ*_*i*_ = 1. Similarly, we get

$$\begin{array}{c} signe_{i}\left(t\right)\left({\overset{`}{c}}_{ij}\left(t\right)-{\overset{´}{c}}_{ij}\left(t\right)\right)f_{j}(\Phi_{j}^{-1}\left(x_{j}\left(t-\tau_{ij}\left(t\right)\right)\right)\leq \left({\bar{c}}_{ij}-{\underline{c}}_{ij}\right)M_{j}\gamma_{i} \end{array}$$ (23)

and

$$\begin{array}{ccc} & & signe_{i}\left(t\right)\left({\overset{`}{d}}_{ij}\left(t\right)-{\overset{´}{d}}_{ij}\left(t\right)\right)\int_{-\infty }^{t}K_{ij}\left(t-s\right)f_{j}\left(\Phi_{j}^{-1}\left(x_{j}\left(s\right)\right)\right)ds\\ & & \;\leq \left({\bar{d}}_{ij}-{\underline{d}}_{ij}\right)M_{j}\gamma_{i}\int_{-\infty }^{t}K_{ij}\left(t-s\right)ds\leq \left({\bar{d}}_{ij}-{\underline{d}}_{ij}\right)K_{ij}M_{j}\gamma_{i}. \end{array}$$ (24)

On the other hand,

$$\begin{array}{ccc} & & signe_{i}\left(t\right)\left[-\frac{p_{i}}{w_{i}\left(\Phi_{i}^{-1}\left(y_{i}\left(t\right)\right)\right)}\left(\Phi_{i}^{-1}\left(y_{i}\left(t\right)\right)-\Phi_{i}^{-1}\left(x_{i}\left(t\right)\right)\right)\right]\\ & & \;\leq -signe_{i}\left(t\right)\cdot \frac{p_{i}}{{\bar{w}}_{i}}\cdot {\underline{w}}_{i}e_{i}\left(t\right)=-\frac{p_{i}{\underline{w}}_{i}}{{\bar{w}}_{i}}\left|e_{i}\left(t\right)\right|. \end{array}$$ (25)

Furthermore, since $\Phi_{i}^{-1}\left(\cdot \right)$ is strictly monotone increasing, we know that $sign\left(\Phi_{i}^{-1}\left(y_{i}\left(t\right)\right)-\Phi_{i}^{-1}\left(x_{i}\left(t\right)\right)\right)=signe_{i}\left(t\right)$. Then we have

$$\begin{array}{c} signe_{i}\left(t\right)\left[-\frac{q_{i}}{w_{i}\left(\Phi_{i}^{-1}\left(y_{i}\left(t\right)\right)\right)}sign\left(\Phi_{i}^{-1}\left(y_{i}\left(t\right)\right)-\Phi_{i}^{-1}\left(x_{i}\left(t\right)\right)\right)\right]\leq -\frac{q_{i}\gamma_{i}}{{\bar{w}}_{i}}. \end{array}$$ (26)

Calculating the derivatives of *V*_2_(*t*) and *V*_3_(*t*), we get that

$$\begin{array}{ccc} {\dot{V}}_{2}\left(t\right) & = & \sum_{i=1}^{n}\sum_{j=1}^{n}\frac{1}{1-\sigma_{ij}}c_{ij}^{u}l_{j}{\bar{w}}_{j}|e_{j}\left(t\right)|-\sum_{i=1}^{n}\sum_{j=1}^{n}\frac{1-{\dot{\tau }}_{ij}\left(t\right)}{1-\sigma_{ij}}c_{ij}^{u}l_{j}{\bar{w}}_{j}\left|e_{j}\left(t-\tau_{ij}\left(t\right)\right)\right|\\ & & \leq \sum_{i=1}^{n}\sum_{j=1}^{n}\frac{1}{1-\sigma_{ij}}c_{ij}^{u}l_{j}{\bar{w}}_{j}|e_{j}\left(t\right)|-\sum_{i=1}^{n}\sum_{j=1}^{n}c_{ij}^{u}l_{j}{\bar{w}}_{j}\left|e_{j}\left(t-\tau_{ij}\left(t\right)\right)\right| \end{array}$$ (27)

and

$$\begin{array}{ccc} {\dot{V}}_{3}\left(t\right) & = & \sum_{i=1}^{n}\sum_{j=1}^{n}d_{ij}^{u}l_{j}{\bar{w}}_{j}\int_{-\infty }^{0}K_{ij}\left(-s\right)|e_{j}\left(t\right)|ds-\sum_{i=1}^{n}\sum_{j=1}^{n}d_{ij}^{u}l_{j}{\bar{w}}_{j}\int_{-\infty }^{0}K_{ij}\left(-s\right)\left|e_{j}\left(t+s\right)\right|ds\\ & & \leq \sum_{i=1}^{n}\sum_{j=1}^{n}d_{ij}^{u}l_{j}{\bar{w}}_{j}K_{ij}|e_{j}\left(t\right)|-\sum_{i=1}^{n}\sum_{j=1}^{n}d_{ij}^{u}l_{j}{\bar{w}}_{j}\int_{-\infty }^{t}K_{ij}\left(t-s\right)\left|e_{j}\left(s\right)\right|ds, \end{array}$$ (28)

where assumptions *A*_1_ and *A*_6_ have been used.

Therefore,

$$\begin{array}{ccc} \dot{V}\left(t\right) & \leq & \sum_{i=1}^{n}\left(-a_{i}{\underline{w}}_{i}-\frac{p_{i}{\underline{w}}_{i}}{{\bar{w}}_{i}}\right)|e_{i}\left(t\right)|+\sum_{i=1}^{n}\sum_{j=1}^{n}\left(b_{ij}^{u}l_{j}{\bar{w}}_{j}+\frac{1}{1-\sigma_{ij}}c_{ij}^{u}l_{j}{\bar{w}}_{j}+d_{ij}^{u}l_{j}{\bar{w}}_{j}K_{ij}\right)\left|e_{j}\left(t\right)\right|\\ & & +\sum_{i=1}^{n}\left\{\sum_{j=1}^{n}\left[\left({\bar{b}}_{ij}-{\underline{b}}_{ij}\right)M_{j}+\left({\bar{c}}_{ij}-{\underline{c}}_{ij}\right)M_{j}+\left({\bar{d}}_{ij}-{\underline{d}}_{ij}\right)K_{ij}M_{j}\right]-\frac{q_{i}}{{\bar{w}}_{i}}\right\}\gamma_{i}\\ & = & \sum_{i=1}^{n}\left[-a_{i}{\underline{w}}_{i}-\frac{p_{i}{\underline{w}}_{i}}{{\bar{w}}_{i}}+l_{i}{\bar{w}}_{i}\sum_{j=1}^{n}\left(b_{ji}^{u}+\frac{1}{1-\sigma_{ji}}c_{ji}^{u}+d_{ji}^{u}K_{ji}\right)\right]\left|e_{i}\left(t\right)\right|\\ & & +\sum_{i=1}^{n}\left[\sum_{j=1}^{n}\left({\bar{b}}_{ij}-{\underline{b}}_{ij}+{\bar{c}}_{ij}-{\underline{c}}_{ij}+{\bar{d}}_{ij}K_{ij}-{\underline{d}}_{ij}K_{ij}\right)M_{j}-\frac{q_{i}}{{\bar{w}}_{i}}\right]\gamma_{i}. \end{array}$$ (29)

If the conditions in Theorem 1 are satisfied, we have

$$\begin{array}{c} \dot{V}\left(t\right)\leq -\varepsilon \sum_{i=1}^{n}\gamma_{i}, \end{array}$$ (30)

where

$$\begin{array}{c} \varepsilon =\min_{i}\left[\frac{q_{i}}{{\bar{w}}_{i}}-\sum_{j=1}^{n}\left({\bar{b}}_{ij}-{\underline{b}}_{ij}+{\bar{c}}_{ij}-{\underline{c}}_{ij}+{\bar{d}}_{ij}K_{ij}-{\underline{d}}_{ij}K_{ij}\right)M_{j}\right]>0. \end{array}$$

By using the same analysis methods as those in [44], we can prove there exists a constant t* > 0 such that

$$\begin{array}{c} {∥e(t^{*})∥}_{1}=0\;\text{and}\;{∥e(t)∥}_{1}\equiv 0,\;\forall t\geq t^{*}, \end{array}$$ (31)

where *e*(*t*) = (*e*_1_(*t*), *e*_2_(*t*), …, *e*_*n*_(*t*))^*T*^ and ${∥e(t)∥}_{1}=\sum_{i=1}^{n}\left|e_{i}\left(t\right)\right|.$

According to Definition 1, MCGNNs (1) and (8) achieve synchronization in finite time. The proof is completed.

**Remark 2**. In Theorem 1, since the distributed delays in MCGNNs (1) and (8) are unbounded, it is difficult to estimate the settling time t*.

If the delay kernels satisfy

$$\begin{array}{c} K_{ij}\left(t\right)=\left\{ \begin{array}{cc} 1,\; & 0\leq t\leq \beta_{ij},\\ 0,\; & t>\beta_{ij}, \end{array}\right. \end{array}$$ (32)

where *β*_*ij*_ > 0 are constants, *i*, *j* = 1, 2, …, *n*, MCGNN (1) can be written as

$$\begin{array}{ccc} {\dot{\xi }}_{i}\left(t\right) & = & -w_{i}\left(\xi_{i}\left(t\right)\right)[a_{i}\left(\xi_{i}\left(t\right)\right)-\sum_{j=1}^{n}b_{ij}\left(\xi_{i}\left(t\right)\right)f_{j}\left(\xi_{j}\left(t\right)\right)-\sum_{j=1}^{n}c_{ij}\left(\xi_{i}\left(t\right)\right)f_{j}\left(\xi_{j}\left(t-\tau_{ij}\left(t\right)\right)\right)\\ & & -\sum_{j=1}^{n}d_{ij}\left(\xi_{i}\left(t\right)\right)\int_{t-\beta_{ij}}^{t}f_{j}\left(\xi_{j}\left(s\right)\right)ds-I_{i}],\;i=1,2,\ldots ,n. \end{array}$$ (33)

This is the corresponding response system:

$$\begin{array}{ccc} {\dot{\eta }}_{i}\left(t\right) & = & -w_{i}\left(\eta_{i}\left(t\right)\right)[a_{i}\left(\eta_{i}\left(t\right)\right)-\sum_{j=1}^{n}b_{ij}\left(\eta_{i}\left(t\right)\right)f_{j}\left(\eta_{j}\left(t\right)\right)-\sum_{j=1}^{n}c_{ij}\left(\eta_{i}\left(t\right)\right)f_{j}\left(\eta_{j}\left(t-\tau_{ij}\left(t\right)\right)\right)\\ & & -\sum_{j=1}^{n}d_{ij}\left(\eta_{i}\left(t\right)\right)\int_{t-\beta_{ij}}^{t}f_{j}\left(\eta_{j}\left(s\right)\right)ds-I_{i}]+R_{i}\left(t\right),\;i=1,2,\ldots ,n. \end{array}$$ (34)

In fact, MCGNNs (33) and (34) can also achieve finite time synchronization under the controller (10), what is more, the settling time t* can be estimated.

**Corollary 1**. Let assumptions *A*_1_-*A*_5_ hold. If control gains *p*_*i*_ and *q*_*i*_ satisfy

$$\begin{array}{ccc} p_{i} & \geq & -{\bar{w}}_{i}a_{i}+\frac{{\bar{w}}_{i}^{2}l_{i}}{{\underline{w}}_{i}}\sum_{j=1}^{n}\left(b_{ji}^{u}+\frac{1}{1-\sigma_{ji}}c_{ji}^{u}+d_{ji}^{u}\beta_{ji}\right),\\ q_{i} & > & {\bar{w}}_{i}\sum_{j=1}^{n}\left({\bar{b}}_{ij}-{\underline{b}}_{ij}+{\bar{c}}_{ij}-{\underline{c}}_{ij}+{\bar{d}}_{ij}\beta_{ij}-{\underline{d}}_{ij}\beta_{ij}\right)M_{j},\;i=1,2,\ldots ,n, \end{array}$$ (35)

MCGNN (34) will be synchronized with MCGNN (33) in finite time under the controller (10). Moreover, the settling time

$$\begin{array}{c} t^{*}\leq \frac{1}{\varepsilon }[\sum_{i=1}^{n}|e_{i}\left(0\right)|+\sum_{i=1}^{n}\sum_{j=1}^{n}\frac{1}{1-\sigma_{ij}}c_{ij}^{u}l_{j}{\bar{w}}_{j}\int_{-\tau_{ij}\left(0\right)}^{0}|e_{j}\left(z\right)|dz+\sum_{i=1}^{n}\sum_{j=1}^{n}d_{ij}^{u}l_{j}{\bar{w}}_{j}\int_{-\beta_{ij}}^{0}\int_{s}^{0}\left|e_{j}\left(z\right)\right|dzds], \end{array}$$

where

$$\begin{array}{c} \varepsilon =\min_{i}[\frac{q_{i}}{{\bar{w}}_{i}}-\sum_{j=1}^{n}\left({\bar{b}}_{ij}-{\underline{b}}_{ij}+{\bar{c}}_{ij}-{\underline{c}}_{ij}+{\bar{d}}_{ij}\beta_{ij}-{\underline{d}}_{ij}\beta_{ij}\right)M_{j}]>0. \end{array}$$

**Proof**. Consider such a Lyapunov function:

$$\begin{array}{c} V\left(t\right)=V_{1}\left(t\right)+V_{2}\left(t\right)+V_{3}\left(t\right), \end{array}$$ (36)

where

$$\begin{array}{ccc} V_{1}\left(t\right) & = & \sum_{i=1}^{n}\left|e_{i}\left(t\right)\right|,\\ V_{2}\left(t\right) & = & \sum_{i=1}^{n}\sum_{j=1}^{n}\frac{1}{1-\sigma_{ij}}c_{ij}^{u}l_{j}{\bar{w}}_{j}\int_{t-\tau_{ij}\left(t\right)}^{t}\left|e_{j}\left(z\right)\right|dz,\\ V_{3}\left(t\right) & = & \sum_{i=1}^{n}\sum_{j=1}^{n}d_{ij}^{u}l_{j}{\bar{w}}_{j}\int_{-\beta_{ij}}^{0}\int_{t+s}^{t}\left|e_{j}\left(z\right)\right|dzds. \end{array}$$ (37)

Referring to the proofs of Theorem 1 and Ref. [44], we can give the remaining proof of Corollary 1, which is omitted here.

**Remark 3**. In MCGNN (1), if $\int_{-\infty }^{t}K_{ij}\left(t-s\right)f_{j}\left(\xi_{j}\left(s\right)\right)ds$ is replaced by $\int_{t-\rho_{ij}\left(t\right)}^{t}f_{j}\left(\xi_{j}\left(s\right)\right)ds$, where 0 ≤ *ρ*_*ij*_(*t*) ≤ *ρ*_*ij*_, we can get a new MCGNN. Similarly to Corollary 1, we can prove that MCGNNs with this kind of distributed time-varying delays can achieve finite time synchronization under the controller (10), and the settling time t* can also be estimated.

**Remark 4**. It has been proved that controller (10) can synchronize MCGNNs effectively. Controller (10) consists of two parts: linear part −*p*_*i*_(*η*_*i*_(*t*) − *ξ*_*i*_(*t*)) and nonlinear part −*q*_*i*_*sign*(*η*_*i*_(*t*) − *ξ*_*i*_(*t*)). In the proofs of Theorem 1 and Corollary 1, the nonlinear part of the controller is used to deal with the parameter mismatches of the drive-response MCGNNs, while the linear part of the controller plays a key role in driving the response MCGNN to synchronize with the drive MCGNN.

**Remark 5**. In [43], the authors also investigated the finite time synchronization of MCGNNs. However, the finite-time synchronization analysis methods they utilized were traditional ones [50], that is, they should prove $\dot{V}\left(t\right)\leq -\alpha V^{\eta }\left(t\right)$, *α* > 0, 0 < *η* < 1, or $\dot{V}\left(t\right)\leq -\alpha V^{\eta }\left(t\right)+\theta V\left(t\right)$, *α* > 0, *θ* > 0, 0 < *η* < 1, where *V*(*t*) is the Lyapunov function. In this paper, we utilize some novel finite-time synchronization analysis methods [44]. First, we prove that $\dot{V}\left(t\right)\leq -\varepsilon \sum_{i=1}^{n}\gamma_{i}$, where *ε* > 0; *γ*_*i*_ = 0 if *e*_*i*_(*t*) = 0, otherwise *γ*_*i*_ = 1. Then we use the strict mathematic analysis to derive the results. Moreover, though the delays considered in [43] were only discrete delays, the controller used in [43] was very complicated, i.e. $R_{i}\left(t\right)=-p_{i}\left(v_{i}\left(t\right)-u_{i}\left(t\right)\right)-\eta_{i}sign\left(v_{i}\left(t\right)-u_{i}\left(t\right)\right)-\sum_{j=1}^{n}k_{ij}sign\left(v_{j}\left(t\right)-u_{j}\left(t\right)\right)-\sum_{j=1}^{n}\delta_{ij}sign\left(v_{i}\left(t\right)-u_{i}\left(t\right)\right)\left|v_{j}\left(t-\tau_{j}\left(t\right)\right)-u_{j}\left(t-\tau_{j}\left(t\right)\right)\right|$. In this paper, we consider MCGNN model with mixed delays, however, the controller that we use is very simple, i.e. *R*_*i*_(*t*) = −*p*_*i*_(*η*_*i*_(*t*) − *ξ*_*i*_(*t*)) − *q*_*i*_*sign*(*η*_*i*_(*t*) − *ξ*_*i*_(*t*)).

**Remark 6**. In MCGNN (1), if the memristive connection weights *b*_*ij*_(*ξ*_*i*_(*t*)) = *b*_*ij*_, *c*_*ij*_(*ξ*_*i*_(*t*)) = *c*_*ij*_ and *d*_*ij*_(*ξ*_*i*_(*t*)) = 0, MCGNN (1) will reduce into the Cohen-Grossberg neural network model studied in [39, 40]. Therefore, the theoretical results of this paper can be applicable to the Cohen-Grossberg neural networks in [39, 40], while the opposite is probably not true. In this sense, the obtained results of this paper are less conservative.

## Numerical simulations

In this section, numerical simulations are given to validate the obtained results in this paper.

**Example 1**. Consider the following MCGNN:

$$\begin{array}{ccc} {\dot{\xi }}_{i}\left(t\right) & = & -w_{i}\left(\xi_{i}\left(t\right)\right)[a_{i}\left(\xi_{i}\left(t\right)\right)-\sum_{j=1}^{2}b_{ij}\left(\xi_{i}\left(t\right)\right)f_{j}\left(\xi_{j}\left(t\right)\right)-\sum_{j=1}^{2}c_{ij}\left(\xi_{i}\left(t\right)\right)f_{j}\left(\xi_{j}\left(t-\tau_{ij}\left(t\right)\right)\right)\\ & & -\sum_{j=1}^{2}d_{ij}\left(\xi_{i}\left(t\right)\right)\int_{-\infty }^{t}K_{ij}\left(t-s\right)f_{j}\left(\xi_{j}\left(s\right)\right)ds-I_{i}],\;i=1,2, \end{array}$$ (38)

where *a*_1_(*v*) = 1.8*v*, *a*_2_(*v*) = 1.6*v*, *τ*_11_(*t*) = 1 − 0.2*sint*, *τ*_12_(*t*) = 0.9 − 0.1*cost*, *τ*_21_(*t*) = 0.5*sint*, *τ*_22_(*t*) = 0.5*cost*, *I*_1_ = −0.02, *I*_2_ = −0.12, $w_{i}\left(v\right)=1+\frac{0.3}{2+tanh(v)}$, *f*_*j*_(*v*) = *tanh*(*v*), *K*_*ij*_(*t*) = *e*^−0.5*t*^, *i*, *j* = 1, 2, and *Υ*_1_ = *Υ*_2_ = 1, $b_{11}^{\prime }=0.25$, $b_{11}^{\prime \prime }=-0.18$, $b_{12}^{\prime }=-1.2$, $b_{12}^{\prime \prime }=0.95$, $b_{21}^{\prime }=-0.85$, $b_{21}^{\prime \prime }=0.25$, $b_{22}^{\prime }=0.36$, $b_{22}^{\prime \prime }=-0.18$, $c_{11}^{\prime }=0.60$, $c_{11}^{\prime \prime }=0.70$, $c_{12}^{\prime }=-0.24$, $c_{12}^{\prime \prime }=-0.15$, $c_{21}^{\prime }=0.56$, $c_{21}^{\prime \prime }=-0.68$, $c_{22}^{\prime }=0.85$, $c_{22}^{\prime \prime }=0.45$, $d_{11}^{\prime }=-0.56$, $d_{11}^{\prime \prime }=-0.25$, $d_{12}^{\prime }=0.15$, $d_{12}^{\prime \prime }=-0.18$, $d_{21}^{\prime }=0.76$, $d_{21}^{\prime \prime }=0.56$, $d_{22}^{\prime }=-0.85$, $d_{22}^{\prime \prime }=-0.35$.

**Fig 2:**
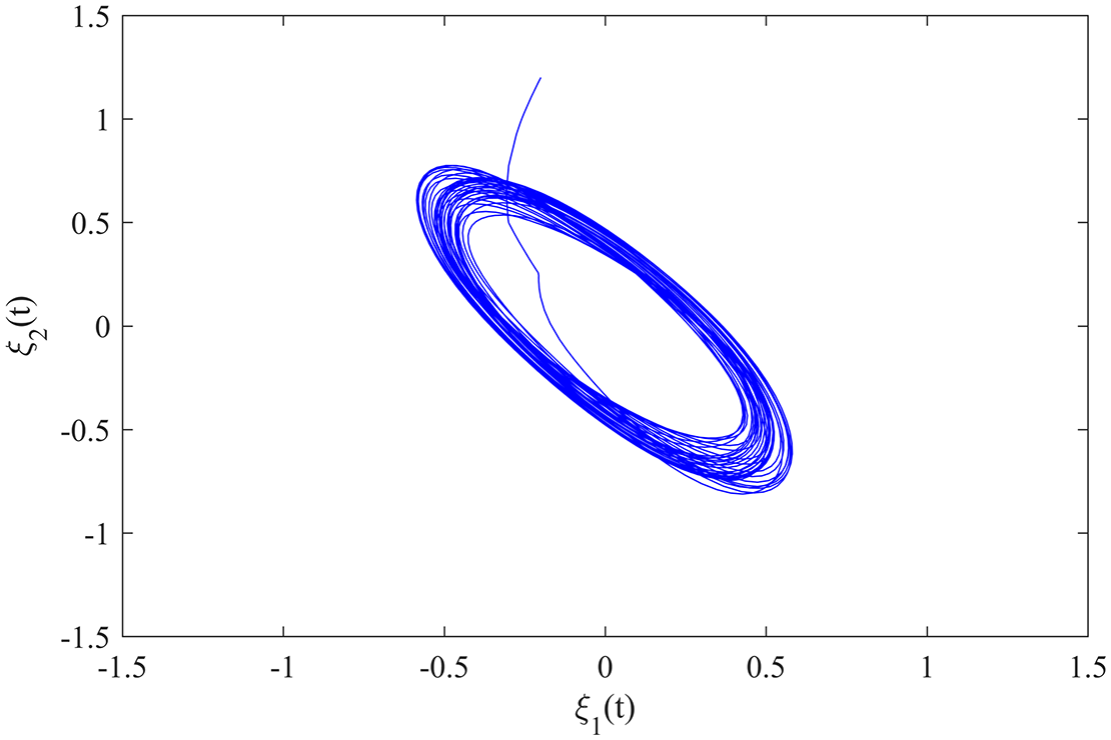
The transient behaviour of MCGNN (38).

The initial value of MCGNN (38) is *φ*(*t*) = (−0.2, 1.2)^*T*^ for *t* ∈ [−5, 0], and *φ*(*t*) = (0, 0)^*T*^ for *t* ∈ (−∞, −5). The transient behaviour of MCGNN (38) is showed in Fig 2.

This is the corresponding response system:

$$\begin{array}{ccc} {\dot{\eta }}_{i}\left(t\right) & = & -w_{i}\left(\eta_{i}\left(t\right)\right)[a_{i}\left(\eta_{i}\left(t\right)\right)-\sum_{j=1}^{2}b_{ij}\left(\eta_{i}\left(t\right)\right)f_{j}\left(\eta_{j}\left(t\right)\right)-\sum_{j=1}^{2}c_{ij}\left(\eta_{i}\left(t\right)\right)f_{j}\left(\eta_{j}\left(t-\tau_{ij}\left(t\right)\right)\right)\\ & & -\sum_{j=1}^{2}d_{ij}\left(\eta_{i}\left(t\right)\right)\int_{-\infty }^{t}K_{ij}\left(t-s\right)f_{j}\left(\eta_{j}\left(s\right)\right)ds-I_{i}]+R_{i}\left(t\right),\;i=1,2. \end{array}$$ (39)

**Fig 3:**
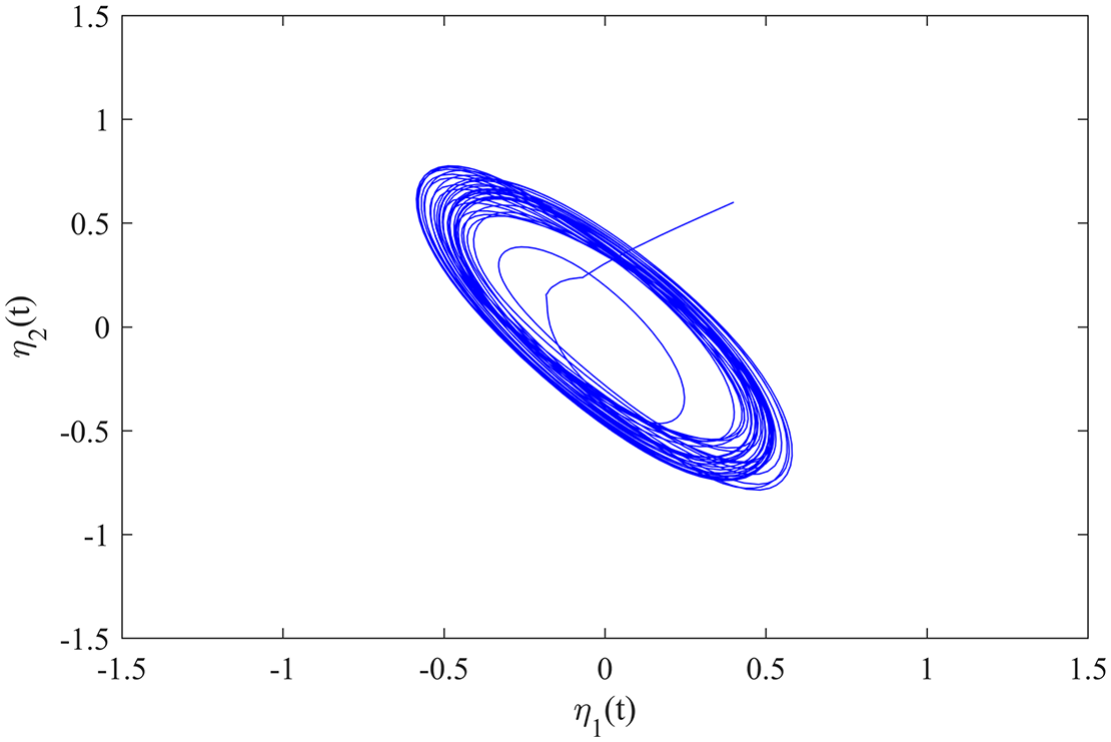
The transient behaviour of MCGNN (39) without control inputs.

The initial value of MCGNN (39) is *ϕ*(*t*) = (0.4, 0.6)^*T*^ for *t* ∈ [−5, 0], and *ϕ*(*t*) = (0, 0)^*T*^ for *t* ∈ (−∞, −5). The transient behaviour of MCGNN (39) without control inputs is showed in Fig 3.

**Fig 4:**
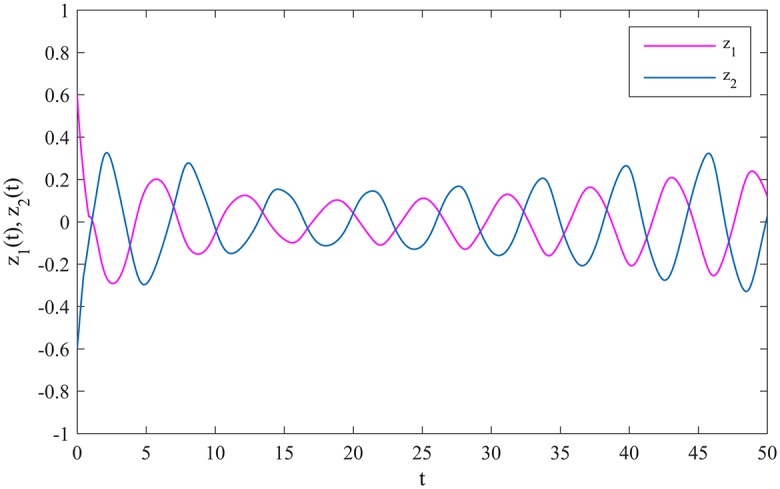
The evolutions of the synchronization errors without control inputs.

The synchronization errors between MCGNNs (38) and (39) are defined as *z*_*i*_(*t*) = *η*_*i*_(*t*) − *ξ*_*i*_(*t*), *i* = 1, 2. The evolutions of the synchronization errors between MCGNNs (38) and (39) without control inputs are showed in Fig 4.

**Fig 5:**
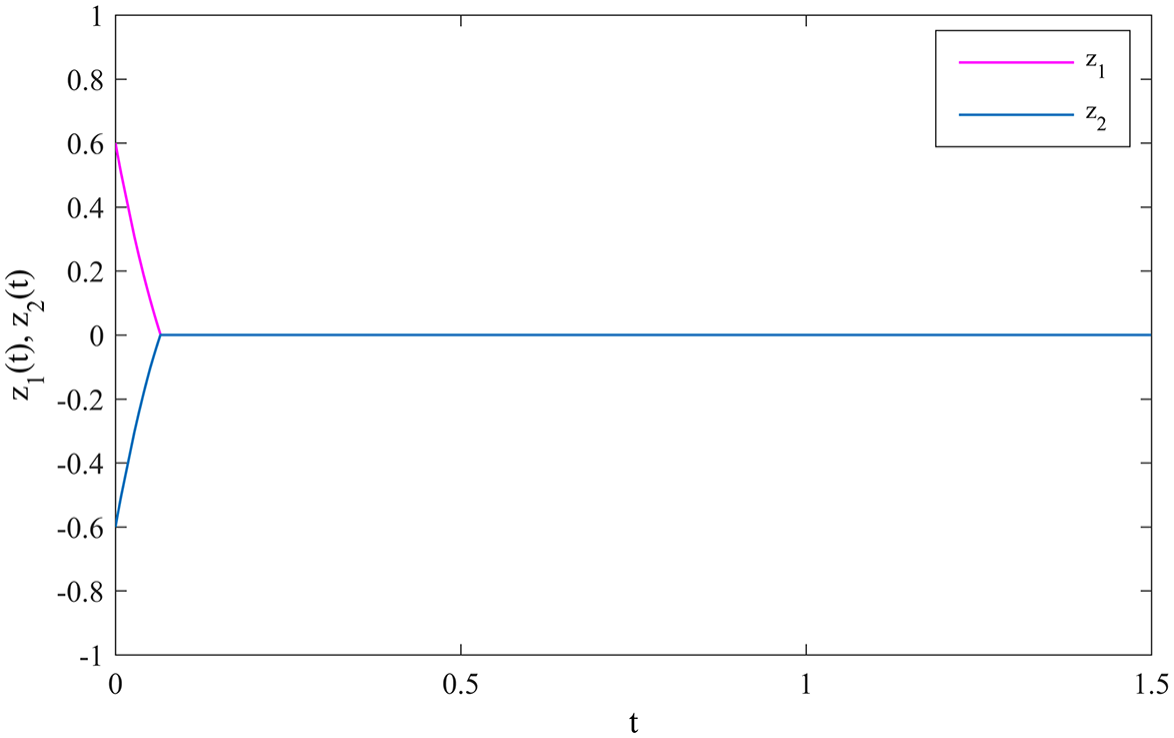
The evolutions of the synchronization errors under the controller (10).

Obviously, *τ*_11_ = 1.2, *τ*_12_ = 1, *τ*_21_ = 0.5, *τ*_22_ = 0.5, *σ*_11_ = 0.2, *σ*_12_ = 0.1, *σ*_21_ = 0.5, *σ*_22_ = 0.5, *a*_1_ = 1.8, *a*_2_ = 1.6, ${\underline{w}}_{i}=1.1$, ${\bar{w}}_{i}=1.3$, *l*_*i*_ = 1, *M*_*i*_ = 1, *K*_*ij*_ = 2, *i*, *j* = 1, 2, so assumptions *A*_1_-*A*_6_ hold. According to Theorem 1, if we choose *p*_1_ = 7, *p*_2_ = 6.6, *q*_1_ = 5.4 and *q*_2_ = 6.2, MCGNN (39) will be synchronized with MCGNN (38) in finite time under the controller (10). Fig 5 shows the evolutions of the synchronization errors between MCGNNs (38) and (39) under the controller (10).

**Fig 6:**
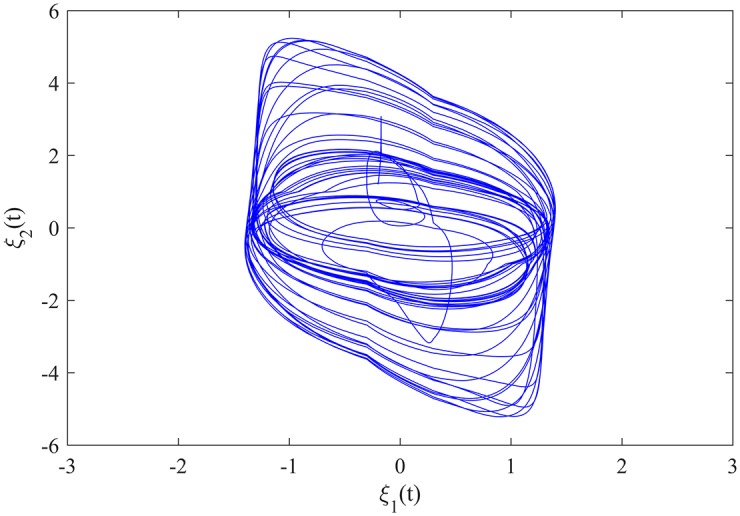
The transient behaviour of MCGNN (40).

**Example 2**. Consider the following MCGNN:

$$\begin{array}{ccc} {\dot{\xi }}_{i}\left(t\right) & = & -w_{i}\left(\xi_{i}\left(t\right)\right)[a_{i}\left(\xi_{i}\left(t\right)\right)-\sum_{j=1}^{2}b_{ij}\left(\xi_{i}\left(t\right)\right)f_{j}\left(\xi_{j}\left(t\right)\right)-\sum_{j=1}^{2}c_{ij}\left(\xi_{i}\left(t\right)\right)f_{j}\left(\xi_{j}\left(t-\tau_{ij}\left(t\right)\right)\right)\\ & & -I_{i}],\;i=1,2, \end{array}$$ (40)

where *a*_1_(*v*) = 1.61*v* + *sin*(*v*), *a*_2_(*v*) = 1.45*v* + *sin*(*v*), *I*_1_ = *I*_2_ = 0, $w_{1}\left(v\right)=6+\frac{1}{1+v^{2}}$, $w_{2}\left(v\right)=3-\frac{1}{1+v^{2}}$, *Υ*_1_ = 0.3, *Υ*_2_ = 1, $b_{11}^{\prime }=1.81$, $b_{11}^{\prime \prime }=2.2$, $b_{12}^{\prime }=-0.14$, $b_{12}^{\prime \prime }=0.12$, $b_{21}^{\prime }=-1.9$, $b_{21}^{\prime \prime }=-2.2$, $b_{22}^{\prime }=5$, $b_{22}^{\prime \prime }=5.2$, $c_{11}^{\prime }=-0.95$, $c_{11}^{\prime \prime }=-1.3$, $c_{12}^{\prime }=0.08$, $c_{12}^{\prime \prime }=0.15$, $c_{21}^{\prime }=-0.2$, $c_{21}^{\prime \prime }=-0.18$, $c_{22}^{\prime }=-2.5$, $c_{22}^{\prime \prime }=-2.3$, and *f*_*j*_(⋅), *τ*_*ij*_(*t*), *i*, *j* = 1, 2, are the same as those in Example 1. The initial value of MCGNN (40) is *φ*(*t*) = (−0.2, 1.2)^*T*^, *t* ∈ [−2, 0]. The transient behaviour of MCGNN (40) is showed in Fig 6.

This is the corresponding response system:

$$\begin{array}{ccc} {\dot{\eta }}_{i}\left(t\right) & = & -w_{i}\left(\eta_{i}\left(t\right)\right)[a_{i}\left(\eta_{i}\left(t\right)\right)-\sum_{j=1}^{2}b_{ij}\left(\eta_{i}\left(t\right)\right)f_{j}\left(\eta_{j}\left(t\right)\right)-\sum_{j=1}^{2}c_{ij}\left(\eta_{i}\left(t\right)\right)f_{j}\left(\eta_{j}\left(t-\tau_{ij}\left(t\right)\right)\right)\\ & & -I_{i}]+R_{i}\left(t\right),\;i=1,2. \end{array}$$ (41)

**Fig 7:**
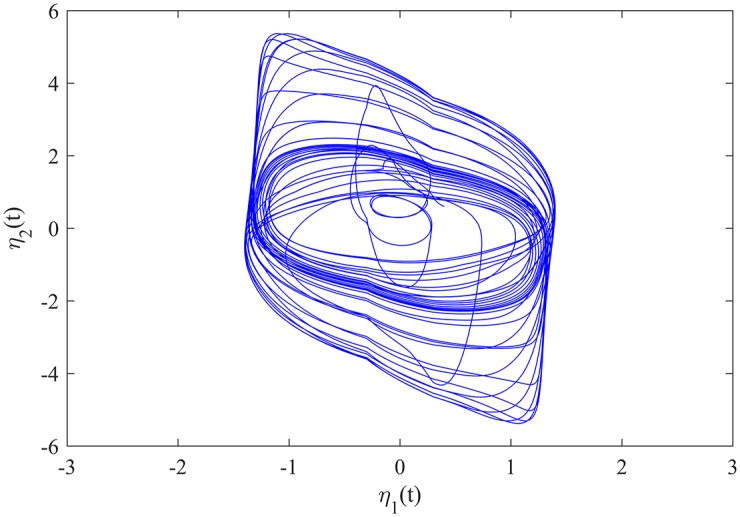
The transient behaviour of MCGNN (41) without control inputs.

**Fig 8:**
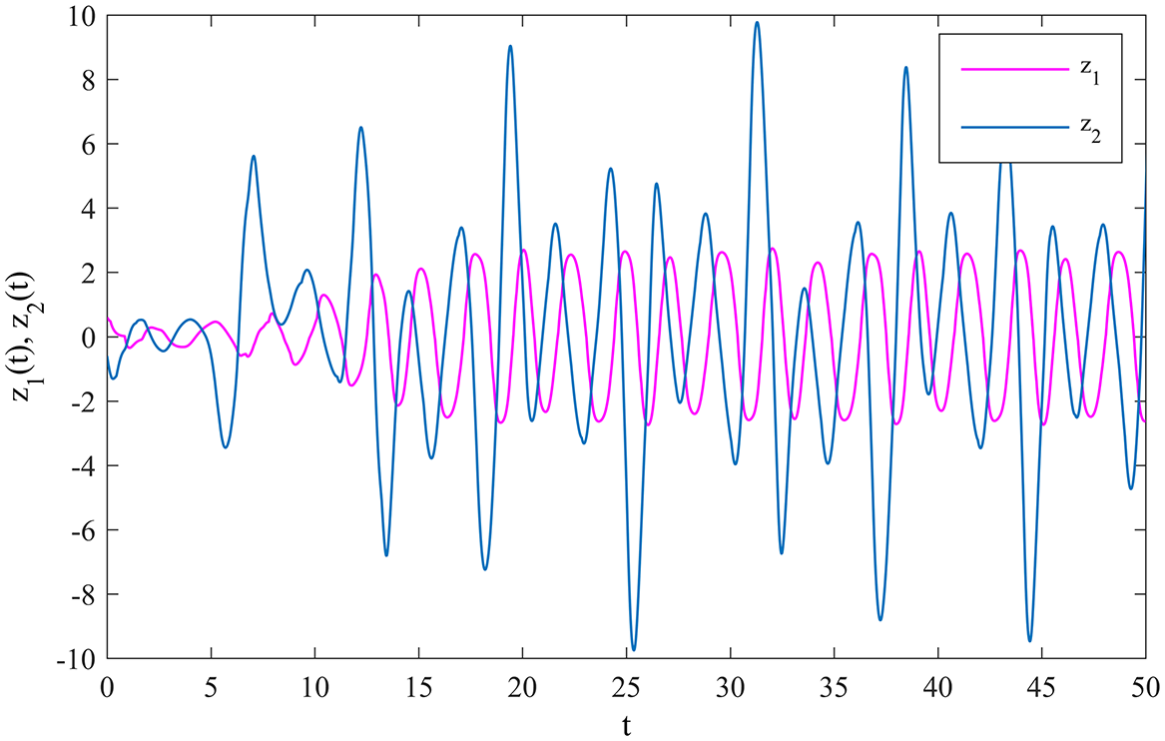
The evolutions of the synchronization errors without control inputs.

The initial value of MCGNN (41) is *ϕ*(*t*) = (0.4, 0.6)^*T*^, *t* ∈ [−2, 0]. The transient behaviour of MCGNN (41) without control inputs is showed in Fig 7. The evolutions of the synchronization errors between MCGNNs (40) and (41) without control inputs are showed in Fig 8.

**Fig 9:**
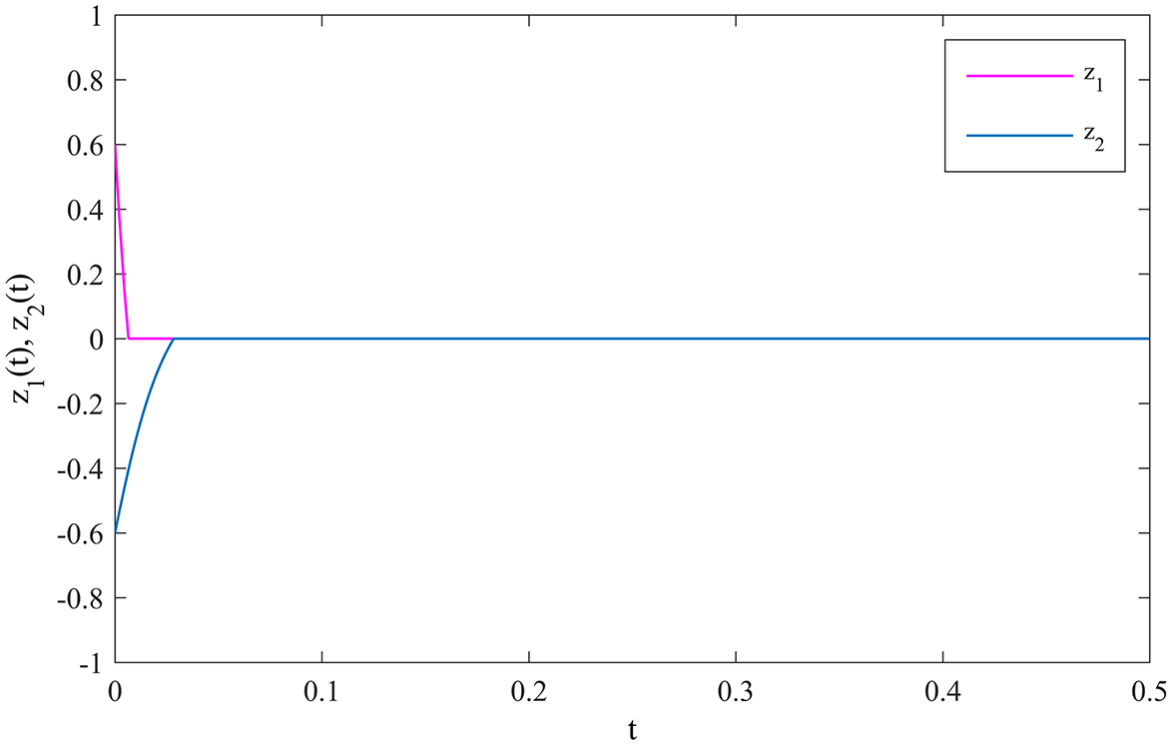
The evolutions of the synchronization errors under the controller (10).

According to Corollary 1, if we choose *p*_1_ = 48.3, *p*_2_ = 45.6, *q*_1_ = 20 and *q*_2_ = 10, MCGNN (41) will be synchronized with MCGNN (40) in finite time under the controller (10), and the settling time t* can be estimated as 7.143. Fig 9 shows that MCGNNs (40) and (41) realize finite time synchronization within t*.

## Conclusion

This paper studies the finite time synchronization problem of MCGNNs with mixed delays. By utilizing some novel and effective analysis techniques, several sufficient conditions that can guarantee the finite time synchronization of MCGNNs with mixed delays are derived. The feedback controllers that we design are very simple, but they can solve the parameter mismatch problem of the drive-response MCGNNs perfectly. However, the conservativeness of the theoretical analysis probably makes the control gains of our feedback controllers much larger than those needed in the engineering applications. On the other hand, it is costly and impractical to control a network by applying controllers to all the nodes. Since adaptive pinning controller can avoid the high control gains effectively and reduce the number of the controlled nodes, our future work will focus on the synchronization control of MCGNNs via the adaptive pinning control. Numerical simulations are given to verify the obtained theoretical results.
